# Biomarkers in Schizophrenia: Current Approaches and New Developments—A Literature Review

**DOI:** 10.1155/bn/2991323

**Published:** 2025-10-15

**Authors:** Alicja Sierakowska, Ewa Niewiadomska, Sebastian Łabuda, Anna Bieniasiewicz, Mateusz Roszak, Beata Łabuz-Roszak

**Affiliations:** ^1^ Institute of Medical Sciences, University of Opole, Opole, Poland, uni.opole.pl; ^2^ Department of Psychiatry, St. Jadwiga Provincial Specialist Hospital, Opole, Poland; ^3^ Department of Epidemiology and Biostatistics, Faculty of Public Health in Bytom, Medical University of Silesia, Katowice, Poland, sum.edu.pl; ^4^ Department of Neurology, St. Jadwiga Provincial Specialist Hospital, Institute of Medical Sciences, University of Opole, Opole, Poland, uni.opole.pl; ^5^ Student Scientific Association at the Department of Neurology, Institute of Medical Sciences, University of Opole, Opole, Poland, uni.opole.pl

**Keywords:** biomarkers, etiopathogenesis, schizophrenia

## Abstract

Schizophrenia (SZ) is categorized as a chronic severe highly heritable brain disease. Symptoms include positive, negative, and cognitive symptoms. Despite numerous theories concerning the etiopathogenesis of SZ, the symptoms, although characteristic in their phenomenology, manifest themselves in a rather heterogeneous manner, which makes them subject to clinical assessment and, at the same time, prone to errors resulting from diverse interpretations of the context of the patient′s statements. Therefore, current research is focusing on identifying more subtle and stable features of SZ, such as the phenotype, endophenotype, and assessable abnormalities devoid of human clinical observation. The various biomarker developments focus on the role of transmitters and their corresponding receptors, in particular: glutamate, acetylcholine, dopamine, or serotonin. Also important in terms of etiopathogenesis remain growth factors such as brain‐derived neurotrophic factor (BDNF), nerve growth factor receptor (NGFR), or vascular endothelial growth factor (VEGF). More recently, research has emphasized the role of inflammatory processes and secreted pro‐ as well as anti‐inflammatory cytokines, included in the class of interleukins, chemokines, and tumor necrosis factors, as well as on inflammatory markers—C‐reactive protein (CRP) or glutathione (GSH). Increasingly, changes at the genetic level have been implicated as the cause of diseases, and it is now believed that noncoding RNAs (micro‐RNA [miRNA], long noncoding RNA [lnc‐RNA], and circular RNA [circRNA]) are involved in the development of SZ. Among the genes that may prove to be potential biomarkers in SZ belong SEDT1A, FOXP2, GRIN2A, GRIA3, NRN1, BDNF, CACNA1C, and ZNF8A4. The peptide group molecules, Phospholipase A2, Klotho protein, and soluble urokinase plasminogen activator receptor (suPAR), also remain consistently important. From the perspective of SZ as a disease associated with neuronal damage, biomarkers correlating with brain injury, neuron‐specific enolase (NSE), and S100B protein should be considered.

## 1. Introduction

Schizophrenia (SZ) is categorized as a chronic, severe, highly heritable brain disease. Symptoms include positive, negative, and also those associated with cognitive impairment. Positive symptoms include disturbances in content and thinking, as well as perceptual disturbances (mainly hallucinations as well as pseudohallucinations). Negative symptoms include apathy, avolition, anhedonia, autism, anergy, and “ambi‐” phenomena. Cognitive deficits present in the form of impaired working memory functioning, attention, and abstract thinking [1]. The underlying cause of SZ can be seen in brain pathology at the subcortical and cortical levels. Numerous reports from the fields of genetics, pharmacology, and postmortem studies support the hypothesis that dopamine regulation disorders are the underlying cause of SZ [2, 3]. Furthermore, all currently approved antipsychotic drugs act on dopaminergic receptors [4]. Neuroimaging techniques have demonstrated increased dopamine synthesis and release in the striatum in patients with SZ [5, 6]. Furthermore, the greatest increase in dopamine is observed in areas of the striatum connected to regions of the frontal cortex [5], while the increase in dopamine release remains positively correlated with the severity of psychotic symptoms [7] and the severity of prodromal symptoms of SZ [8]. Dopamine regulation disorders in the striatum are also indicated by the aberrant salience hypothesis, according to which abnormal neurotransmitter secretion in this region is responsible for misinterpretations of the significance of irrelevant stimuli, which determines the development of psychosis [9]. At the cortical level of the brain, abnormalities are primarily found in the functioning of neural networks. According to current knowledge, it is suggested that activity in the dorsal attention network (DAN) and frontoparietal network (FPN) is associated with the allocation of attention to external stimuli. In contrast, activity in the default mode network (DMN) correlates with the regulation of attention to internal representations, thereby determining the normal course of wakefulness. According to the above theory, tasks that require focusing attention on external stimuli result in increased DAN/FPN activity and decreased DMN activity, and vice versa [10]. Therefore, dysregulation of the balance between DAN/FPN and DMN is responsible for the occurrence of cognitive disorders, as well as processing deficits such as cognitive inefficiency or impaired context processing [11]. In 2017, brain networks were identified—cortical midline structures (CMS)—that are the basis of self‐referential processing [12], within which increased activity was observed during the occurrence of self‐referential delusions in patients [12]. Hyperactivity in the networks responsible for speech perception (SP) was identified as the source of auditory verbal hallucinations (AVHs) [13]. During SP processes, but not in the case of auditory oddball (AO) or verbal thought generation (VTG), increased activity and, at the same time, abnormal functioning of the DMN network are observed in patients with AVH [14]. From the perspective of evidence integration, which is a fundamental process in the formation of delusions, abnormalities should be sought in the form of hyperactivity of the visual attention network (VsAN) and DMN, as well as reduced activation of the cognitive evaluation network (CEN) [15]. Current research is focusing on identifying more subtle and stable features of SZ, such as the phenotype, endophenotype, and assessable abnormalities devoid of human clinical observation. To this end, it seems reasonable to search for and use biomarkers that can be measured from readily available body fluids such as blood serum, cerebrospinal fluid, or urine [16]. Their use would affect not only the precise diagnosis but also the prognosis and monitoring of therapy [17]. SZ biomarkers are divided into those occurring peripherally and centrally. However, due to the systemic nature of the disease, some substances present in the brain area of patients show a correlation with their serum concentrations, as the central nervous system (CNS) and peripheral structures remain related [18]. Biomarkers include those related to the neuroimmune system, those corresponding to neurotransmitter metabolism, those associated with neurotrophic factors, those of genetic origin, and those indicating brain damage [19]. In this review, the authors presented the state of the art in terms of the availability and possible use of biomarkers from the perspective of diagnosis, assessment of prognosis, or response to treatment in SZ disorder. Particular attention was paid to substances that have become of major interest to researchers in recent years.

## 2. Materials and Methods

**Figure 1:**
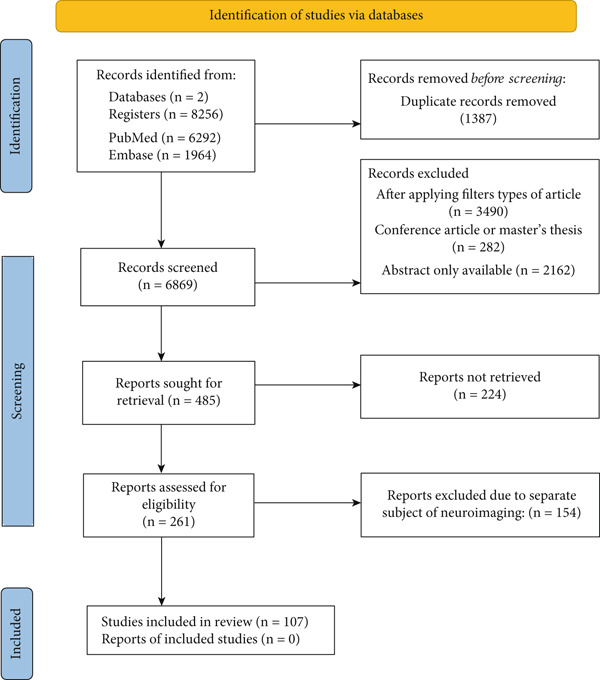
The source selection presented as the PRISMA flow diagram [20]. The date for conducting the analysis is November 1, 2024–January 6, 2025.

A systematic literature review was performed using PubMed and Embase databases. Keywords such as “biomarkers in schizophrenia” OR “markers in schizophrenia” OR “biomarkers in mental disease” OR “markers in mental disease” OR “psychosis” were used during the literature search (*n* = 8256). One thousand three hundred eighty‐seven results overlapped in the databases used. However, 485 papers were finally selected after applying the filters (“clinical trial,” “meta‐analysis,” “randomized control trial,” and “systematic review”) and and the availability of the entire manuscript. Due to the broad topic of the work, publications on neuroimaging were not considered, resulting in the rejection of 154 items. Next, the literature was analyzed, and 107 items relevant to the subject of the study were selected. The detailed process of source selection is presented in the form of a graph (Figure 1).

### 2.1. Biomarkers Related to Neurotransmitters and Their Receptors

One of the key excitatory neurotransmitters that plays an important role in the pathogenesis of SZ is glutamate, whose hyperactivity and excitotoxicity cause the destruction of neural networks [21]. On the other hand, studies show that patients suffering from SZ exhibit reduced mRNA expression for the activity of the xc‐SLC3A2 and SLC7A11 subunits, known as xCT cystine–glutamate antiporters, which regulate the transport of cystine into the cell and glutamate into the extracellular space, while also indicating a decrease in glutamine in patients with SZ [22]. In addition, an association between decreased regulation of the xc‐ system and lower concentrations of the indicated neurotransmitter in the extracellular space has been demonstrated [23]. Determining the expression of systemic xc‐ genes in white blood cells from patients with SZ may facilitate a better understanding of the disorder and contribute to the development of molecular diagnostics [24]. Receptors for glutamate include the N‐methyl‐D‐aspartate receptor (NMDAR) [25]. D‐Serine is a potent coagonist of NMDAR, so its concentration in the brain has remained an object of interest for researchers [26]. The aim of one study was to determine the concentration of substances in people diagnosed with SZ and to compare their values with those of healthy control. It was found that the level of D‐serine in both the cerebrospinal fluid and blood of patients with SZ is lower than in the control group [27, 28]. Furthermore, the presence of clozapine (an antipsychotic drug) increases in plasma D‐serine levels [20]. The results also point to the possible antidepressant properties of D‐serine, making the substance relevant from the perspective of not only SZ but also depression [29]. As is well known, prior to the introduction of modern antipsychotics, mainly targeting the D2 receptor, substances affecting the cholinergic system were used to treat SZ [30]. However, given the numerous side effects of the indicated substances, their clinical usefulness remains limited. On the other hand, the literature reports that abnormalities in acetylcholine levels are associated with the occurrence of negative symptoms as well as cognitive impairment in SZ. Moreover, the additional use of acetylcholinesterase inhibitors in patients with SZ results in a reduction of negative symptoms and also an overall improvement in cognitive function [31]. Other studies report significant improvements in cognitive functioning after discontinuation of medications that reduced acetylcholine availability in SZ sufferers [32]. The response to auditory mismatch negativity (MMN) seems to play an important role as a biomarker of SZ. It correlates with NMDA receptor dysfunction. A significant association between the drug used and MMN was found. Substances that act as NMDAR antagonists delayed and altered the topography of the mismatch response, whereas in the case of dopamine receptor antagonists (amisulpride), no such effect was observed. Differential MMN sensitivity remains associated with muscarinic receptor stimulation [33]. It has been suggested that muscarinic receptor dysfunction (stimulated by acetylcholine) plays an important role in the etiopathogenesis of SZ. Despite the small number of papers investigating muscarinic receptor antibodies, they unilaterally indicate the presence of the described proteins in patients with SZ. These antibodies may represent a biomarker of a hitherto undescribed pathophysiological process of SZ [34]. Serotonin is a tryptophan derivative, synthesized in the sutural nuclei of the brain. According to the serotonin theory of the development of SZ [35], the occurrence of the disease may be related to reduce concentrations of the neurotransmitter in the brain. However, due to its presence only in the CNS, there is difficulty in identifying and at the same time determining the biomarker in blood serum. Metabolism is made possible by the enzymatic activity of monoamine oxidase (MAO), which is responsible for converting serotonin to 5‐hydroxyindoleacetic acid (5HIAA). Due to its penetration into the cerebrospinal fluid, the acid can be used to analyze the concentration of the neurotransmitter in the CNS [36].

### 2.2. Biomarkers Belonging to the Group of Growth Factors

Recently, research on SZ has oscillated around brain‐derived neurotrophic factor (BDNF), due to the important role of neurotrophin in neurogenesis (including serotonergic pathways, glial cells, and hippocampal structures [37, 38]) and synaptogenesis. In addition, the substance′s importance in the neuronal function of the dopaminergic economy has been demonstrated, suggesting the involvement of BDNF in the etiology of SZ [39]. BDNF has also been shown to play a role in long‐term potentiation (LTP), conditioning the learning process [40]. Given the irreversibility of neuron loss, repair, regeneration, and synaptic plasticity processes are considered particularly important from the perspective of neurological and mental disease development [41]. BDNF plays a special role in maintaining normal synaptic transmission, as it regulates all three aspects of their physiology, determining the protection and repair of existing synapses and initiating the formation of new synaptic connections [41]. The molecule is also involved in the regulation of nerve cell function in the limbic system by controlling the expression of D2 and D3 receptors [42]. Serotonergic metabolism, in which abnormalities correlate with the onset of SZ, is also regulated by BDNF. It promotes the development and differentiation of serotonin neurons, the formation of dendritic spines, and synaptic connections [43, 44]. In a study conducted by Penadés et al., the aim was to assess the value of BDNF concentrations and to investigate polymorphism in the gene encoding the factor for a population of patients with a diagnosis of SZ. The experiment involved 70 participants enrolled in the study group and 15 healthy volunteers. The population was assessed for BDNF concentrations before and after treatment implementation, level of cognitive function, and quality of life. Based on the results, there were no significant differences in BDNF values between the groups [45]. The results presented do not remain consistent with other reports. In the paper of González‐Ortega et al., where BDNF was measured in the plasma of 86 patients with a first episode of psychosis, before as well as after the implemented treatment (electroconvulsive treatments, omega‐3 unsaturated fatty acids, and cognitive behavioral therapy [CBT]), an increase in BDNF levels was observed in correlation with a decrease in the severity of psychotic symptoms [46]. The inconclusiveness of the results presented demonstrates the need for further research. The neurotrophins described show affinity for the p75 receptor, known as the nerve growth factor receptor (NGFR) [47]. The name p75 describes its molecular weight. The presence of the receptor has been observed primarily in the developing brain, where it conditions axon pruning [48]. In addition, its activity has been reported in the peripheral nervous system, where it is involved in myelination processes [49]. It has been demonstrated that BDNF regulates neuronal apoptosis through the activation of p75NTR [50]. It has also been proven that the precursor of NGF, proNGF, exhibits greater activity than its mature form, NGF, in the process of inducing apoptosis after binding to the p75NTR subunit [51]. Similar relationships apply to the activity of proBDNF, which is an apoptotic ligand that induces cell death at concentrations lower than normal. However, the action of proBDNF depends on the coexpression of p75NTR and the coreceptor sortilin, which is why neurons deficient in p75NTR and sortilin are resistant to proBDNF‐induced apoptosis [52]. The described receptor has been on the spectrum of interest of numerous researchers. Chen et al. assessed the cleavable extracellular domain (ECD) of the p75NTR receptor in serum samples obtained from 34 participants with SZ and healthy controls. Values of p75NTR ECD concentrations were significantly lower in SZ patients compared to healthy subjects [53]. In another study by He et al., p75NTR ECD determinations were made from patients with a diagnosis of SZ (with a first recorded psychotic episode), individuals at high clinical risk of developing SZ in the future and healthy participants—unencumbered by SZ risk factors constituting the control group. In the first group, the concentration values of the molecule were higher compared to the results from the control group. Nevertheless, they remained lower in relation to participants at risk of developing SZ. Based on the results presented, the authors suggested that neurodegenerative processes may be more severe in the prodromal phase of SZ compared to the full‐blown clinical manifestation of the disease [54]. However, recent studies have presented different results. Zakowicz et al. compared serum p75NTR ECD levels from adolescents with SZ versus healthy subjects. There was no significant difference in the levels of the substance between the groups either on admission to hospital or after 6–8 weeks of treatment. Nevertheless, a correlation of the molecule level with the age of the subjects at which suicidal thoughts first occurred was demonstrated, suggesting the promising potential of using p75NTR ECD as a biomarker of the course of and response to treatment in patients with SZ [55]. Vascular endothelial growth factor (VEGF), associated with angiogenesis, is involved in the regulation of cerebral blood volume and flow. The literature reports that modification of cerebral blood circulation due to changes in the VEGF system affects cognitive performance and brain function in people with ED [56]. However, the analysis of the data on the role of VEGF in the modulation of the angiogenic response in SZ presents controversial results, as some reports advocate elevated serum levels of VEGF in patients, while other authors have not demonstrated such a relationship [57].

### 2.3. Genes as Potential Biomarkers of SZ

Genome‐wide association studies (GWASs) related to SZ have identified over 200 gene variants indicating an increased risk of developing this disease [58]. In this article, the authors present selected genes according to the time criterion included in the reports. Studies indicate that heterozygosity of SEDT1A (a gene that determines the histone methyltransferase process, as well as dendrite development and neuronal metabolism) causes diverse transcriptional adaptations in nerve cells in the prefrontal cortex (PFC) and striatum [59]. FOXP2+ neurons, present in the PFC (activated by the FOXP2 gene in the event of its dysregulation), are responsible for motor control, sound processing, language planning, and learning processes. It is suggested that changes resulting from reduced trimethylation of histone H3 Lysine 4 (associated with SEDT1A expression) result in insufficient development of FOXP2 neurons and, at the same time, predispose to the onset of SZ [60]. As already mentioned in this paper, NMDA receptor hypofunction is at the root of SZ. Recent reports suggest that the GRIN2A gene, which encodes the GluN2A protein (a subunit of NMDAR), is also associated with SZ pathology [61]. Most alleles of the GRIN2A gene cause a shortening of the encoded GluN2A protein and, at the same time, a loss of NMDAR function, which significantly increases the risk of developing SZ [62]. The GRIA3 gene is considered one of the six genes with the highest risk of developing SZ [63]. GRIA3 encodes the GluA3 subunit of the glutamatergic AMPA (*α*‐amino‐3‐hydroxy‐5‐methyl‐4‐isoxazolepropionic acid) receptor. Mutations in this gene can cause a decrease in glutamatergic signaling, which is characteristic of individuals with SZ [64]. Furthermore, a decrease in GRIA3 expression with coexisting Xpo7 haploinsufficiency (a gene involved in nuclear protein transport) increases the risk of SZ more than 20‐fold [65]. The NRN1 gene, responsible for the expression of a neurotrophic factor that plays a key role in the development of the nervous system and synaptic plasticity, is supported by BDNF. NRN1 also contributes to the activation of the insulin receptor by modifying the transcription of the alpha1 C subunit of the voltage‐dependent calcium channel (CACNA1C) [66]. The three genes indicated (NRN1, BDNF, and CACNA1C), which are correlated with each other, have been independently associated with the risk of SZ. In particular, the interaction between individual alleles of NRN1 and BDNF influences the severity of SZ symptoms and morphological changes in the brain, such as the volume of the frontal, parietal, and temporal cortex in patients [67]. One of the genes important for the development of mental illness is the zinc protein gene (ZNF804A), which is associated with the occurrence of SZ, but also bipolar disorder (BD), autism spectrum disorders (ASDs), and anxiety disorders [68]. The gene is located in the cytoplasm of nerve cells in the cerebral cortex, occurring in various types of neurons, in particular those with dopaminergic and GABAergic activity, as well as in pyramidal cells [69]. Furthermore, research results indicate a key role of the ZNF8A4 gene in the development of axons and synapses in the postmitotic period, which is essential for the pathology of neurodevelopmental disorders [69].

### 2.4. Neuropeptide Oligopeptides

In recent years, the importance of neuropeptides as well as oligopeptides as potential markers of nervous system diseases, including SZ, has received increased attention [70]. This fact is related to their ability to modulate the signaling of individual neurotransmitters present in the CNS [70]. Importantly, these enzymes may play a role in neurogenesis, neural cell outgrowth, or neuronal migration. In the following text, proteins relevant to the last decade will be presented [70]. Phospholipase A2 is involved in many physiological activities. For example, plasma calcium–independent Phospholipase A2 (iPLA2) is involved in brain developmental processes such as cortical development, synaptogenesis, glutamate receptor regulation, neuroplasticity, or neurodegenerative processes [71]. Cytosolic Phospholipase A2 (cPLA2) isoform, on the other hand, in addition to its role in neurodegenerative processes, has an important function in neurotransmitter release, as well as learning and memory processes [72, 73]. Reports indicate that people with SZ present abnormal PLA2 values, as well as lower sensitivity to niacin. Yang et al. conducted a study on a Chinese population with the goal of determining the differences in iPLA2 and cPLA2 concentrations between individuals with SZ and healthy participants [74]. The results indicated significantly higher plasma iPLA2 levels in the clinical group, while no differences were observed for cPLA2 concentration values between the groups. Furthermore, a positive correlation was observed between iPLA2 levels and the intensity of skin redness within 20 min [74]. Another representative peptide is the Klotho protein. Until now, its reduction has been associated with bone disease, cardiac problems, or cognitive impairment. However, a role for Klotho in neuropsychiatric disorders has recently been demonstrated. As is known, SZ is characterized by positive symptoms, negative symptoms, and cognitive impairment [75]. In addition, according to neuroinflammatory theory, during SZ, there is a redox imbalance and, at the same time, the occurrence of oxidative stress [76]. Furthermore, SZ is characterized by reduced synthesis of myelin by oligondendrocytes and consequent cognitive impairment [77, 78]. Studies indicate that Klotho protein affects the reduction of neuronal oxidative stress by reducing free radicals. Furthermore, it induces oligodendrocyte maturation, suggesting its key role as a biomarker for SZ [78]. Ubiquitination as well as deubiquitination plays an important role in the regulation of cellular processes; their disruption may underlie various diseases. The proteins involved in the processes described are included in the ubiquitin‐specific peptidase (USP) family. USP53 is widely expressed in human tissues and plays a role in cell apoptosis, neural transmission, or bone remodeling. Therefore, mutation in the USP53 gene may contribute to cholestasis, deafness, and also SZ [79]. José et al. described a USP53 variant (p.Cys228Arg) underlying SZ [80]. The abnormal protein is implicated in ubiquitin processing, folic acid metabolism, and tight junction pathophysiology, which has been linked to the etiopathogenesis of SZ [81]. Soluble urokinase plasminogen activator receptor (suPAR) is categorized as a marker of inflammation [82]. Its presence in numerous body fluids such as blood serum, cerebrospinal fluid, saliva, and urine give it an advantage over other known markers of inflammatory processes [83]. suPAR is resistant to freezing and thawing processes and is stable regardless of the patient′s diet, diurnal rhythm, or body mass index (BMI), which guarantees its reliability as a biomarker [84]. In a study on the use of suPAR in the diagnosis of SZ, it was shown that patients with SZ presented statistically significantly higher levels of serum suPAR values compared to the control group [85, 86].

### 2.5. Biomarkers Associated With Neural Tissue Damage

S100B was described in the 1960s and is classified as a calcium‐binding protein. It is characterized by its complete solubility in ammonium solution—hence its original name is S100 protein [87]. In the nervous system, the highest concentration is observed in astrocytes, but it is also present in other glial cells such as oligodendrocytes, Schwann cells, lining cells, or Muller′s retina [88]. Elevated S100B concentrations in cerebrospinal fluid and blood serum may indicate the presence of a pathological process in the nervous system and are therefore considered reliable biomarkers of this process. Abnormal levels of the described protein may indicate the presence of acute brain injury (ABI), neurodegenerative diseases, perinatal disorders, or psychiatric diseases, including SZ [89]. The current results of available studies on S100B values in patients with SZ are consistent, showing higher protein concentrations in people with SZ compared to healthy participants [90]. The study by Green et al. assessed S100B levels in cerebrospinal fluid in people with SZ. The observed increase in values in the clinical group compared to the control group indicated increased blood–brain barrier (BBB) permeability during the disease [91]. In addition, S100B has found use in the clinical assessment of patients with ABI [92, 93] and, more recently, for mild traumatic brain injury [94]. Neuron‐specific enolase (NSE) is also a marker used in the prognosis of brain injury. This enzyme belongs to a group of enolases that play an important role in the glycolytic pathway. Expression of NSE occurs primarily, during late neuronal differentiation, which determines specificity for neurodevelopmental cells [95]. NSE plays an important role as a marker of ongoing neurodegenerative processes, as well as some neurological or psychiatric conditions, including SZ [96]. In a study by Liu et al., the aim was to compare NSE levels in patients with first episode schizophrenia (FES), participants with chronic schizophrenia (CSZ), and a control group. The results indicated lower NSE concentrations in the CSZ group compared to participants in the FES and control groups [97]. Similar correlations were observed by Andreou et al., assessing NSE levels in patients with SZ, BD, and willing healthy adolescent and adult participants. Significantly lower NSE concentrations were found with both SZ and BP compared to the control group. In addition, both subgroups showed a negative correlation between biomarker levels and symptom severity [98].

### 2.6. Novel Method of SZ Diagnosis

The rapid development of artificial intelligence (AI), in particular machine learning (ML) and deep learning (DL), increases the chance of improving the accuracy, reliability, and effectiveness of mental illness diagnostics [99]. Currently, experiments with AI in psychiatry are limited, and the algorithms used to assess patients are not yet sufficiently understood [100]. Nevertheless, in recent years, the number of reports on this topic has increased significantly, particularly the combination of AI with available biomarkers or clinical features.

In a study by Javeed et al., a ML method based on handwriting data was presented as a new form of diagnosis for SZ and BD. By identifying statistically significant handwriting characteristics specific to patients with SZ or BP and eliminating data imbalances for artificial neural networks, the proposed model achieves an accuracy rate of 96% in diagnosis [101].

Also, from the perspective of morphology and phenotypic traits, discrete anatomical differences (skull height, mouth width, high palate, or tongue protrusion) can be used in the diagnosis of SZ. In a study by Jeng et al., the authors focused on predicting the risk of SZ based on anatomical differences, using, among other things, the random forest (RF) algorithm, which allowed for the most accurate prediction of the disease among the available statistical models [102].

ML also appears to be helpful in the diagnosis of SZ using electroencephalography (EEG) while the patient is at rest. Using learning algorithms to collect a larger number of diverse EEG recordings, it has become possible to distinguish patients with SZ from the healthy population with an accuracy of up to 94% [103, 104]. In 2023, Grover et al., using previous reports, developed the Schizo‐Net model (based on EEG testing), which allows SZ to be detected with a correct diagnosis probability of 99.84%. This tool uses multimodal DL techniques, which outperform previously used techniques [105]. There are reports indicating the use of AI in the diagnosis of early psychosis. A group of 27 young people diagnosed with early psychosis underwent neuropsychological assessment (taking into account the antipsychotic treatment used) to determine the presence of formal thought disorders (FTDs) as an early symptom of psychotic disorders. Numerous ML models were used, but the highest score (correct classification of 24 out of 27 patients) was obtained with the logistic regression (LR) model [106].

### 2.7. Biomarkers From the Perspective of Children and Adolescents

The group of children and adolescents predisposed to experiencing psychotic episodes may be particularly important in terms of observing biological factors influencing the development of SZ or other mental illnesses. Among adult patients experiencing psychotic episodes or diagnosed with SZ, particular attention is paid to the immunological basis of the disorders. However, the results of studies conducted on adolescents and children often contradict this thesis.

A meta‐analysis published in 2017 included 261 patients diagnosed with early psychosis and 246 healthy participants. In six independent studies that assessed the levels of catalase, glutathione, glutathione peroxidase, and superoxide dismutase (as indicators of antioxidant status or oxidative cell damage), no significant differences were found between the participants in both groups. Nevertheless, qualitative measurements showed a positive correlation between the levels of inflammatory and oxidative markers and abnormal clinical outcomes in patients, particularly from the perspective of neurobiological changes in patients [107]. Another meta‐analysis, published in 2024, covering 38 studies of psychosis in people under the age of 18, suggests an increase in inflammatory processes among children and adolescents experiencing psychotic disorders. This is evidenced by a higher neutrophil‐to‐lymphocyte ratio (NLR) and an increase in tumor necrosis factor (TNF‐B), C‐reactive protein (CRP), and Interleukin 6 (IL‐6) in patients with psychosis compared to the control group [108]. From the perspective of biomarkers belonging to the group of proteins or growth factors, the published results of studies conducted on children and adolescents remain consistent with those concerning the adult population. Based on an experiment conducted on patients with a first episode of SZ (192 participants) and a control group (136 healthy volunteers), lower BDNF levels are suggested among patients in the clinical group. In addition, BDNF levels remained negatively correlated with the total score on the positive and negative symptom scale for SZ [109]. Similar results were obtained by Zakowicz et al. in a study of adolescents diagnosed with SZ [55]. Considering the genes described, characteristics of the pediatric population diagnosed with SZ, the importance of the CUB and Sushi Domains 1 (SCMD1) gene is pointed out. It plays a role in the process of neurogenesis, affects the proper functioning of memory and immunity, and also participates in monoamine metabolism [110]. A study involving children and young adults with a first episode of SZ from families at high risk of SZ and a control group showed lower levels of SMD1 gene mRNA expression and the corresponding protein compared to the healthy population. It is suggested that CSMD1 expression levels may in the future become a reliable biomarker for the first episode of SZ in children and adolescents [111].

## 3. Limitations of the Study

One of the goals of this publication was to collect and present the available knowledge on the various biomarkers that play a role in the diagnosis and treatment of SZ. For this reason, the authors included articles labeled “clinical trial,” “meta‐analysis,” “randomized control trial,” and “systematic review.” The number of available papers made the analysis much more difficult, so neuroimaging reports were excluded from the review, significantly narrowing the search field. It should be noted that some of the studies cited were conducted on a relatively small group of participants, which is due to the continuous development of new theories and, at the same time, the first‐time nature of individual experiments. Nevertheless, the correlations indicated point the way forward for further work on biomarkers.

## 4. Conclusions

In recent years, research into SZ has focused mainly on the search for a reliable biomarker that would enable accurate diagnosis (with a lower risk of error). The biomarkers considered in this paper (related to neurotransmitters, neuroinflammatory processes, neurotrophin, genes, the structures encoding them, or neural tissue damage) have been quite extensively studied in terms of changes in serum concentrations. Nevertheless, based on reports to date, no specific pathophysiological theory of the disease has been detailed to give an advantage over the others. Therefore, further clinical and randomized studies in almost every area presented in this paper are needed to determine exact correlations and, eventually, target substance concentrations, which would allow the diagnosis to be unequivocally confirmed or excluded.

## Conflicts of Interest

The authors declare no conflicts of interest.

## Funding

No funding was received for this manuscript.

## Supporting information


**Supporting Information** Additional supporting information can be found online in the Supporting Information section. PRISMA checklist.

## Data Availability

Data sharing is not applicable to this article as no datasets were generated or analyzed during the current study.
